# Discovery of Novel Inhibitors for Nek6 Protein through Homology Model Assisted Structure Based Virtual Screening and Molecular Docking Approaches

**DOI:** 10.1155/2014/967873

**Published:** 2014-01-22

**Authors:** P. Srinivasan, P. Chella Perumal, A. Sudha

**Affiliations:** Department of Bioinformatics, Alagappa University, Karaikudi, Tamilnadu 630 004, India

## Abstract

Nek6 is a member of the NIMA (never in mitosis, gene A)-related serine/threonine kinase family that plays an important role in the initiation of mitotic cell cycle progression. This work is an attempt to emphasize the structural and functional relationship of Nek6 protein based on homology modeling and binding pocket analysis. The three-dimensional structure of Nek6 was constructed by molecular modeling studies and the best model was further assessed by PROCHECK, ProSA, and ERRAT plot in order to analyze the quality and consistency of generated model. The overall quality of computed model showed 87.4% amino acid residues under the favored region. A 3 ns molecular dynamics simulation confirmed that the structure was reliable and stable. Two lead compounds (Binding database ID: 15666, 18602) were retrieved through structure-based virtual screening and induced fit docking approaches as novel Nek6 inhibitors. Hence, we concluded that the potential compounds may act as new leads for Nek6 inhibitors designing.

## 1. Introduction 

Mitotic errors and misregulation of cell cycle process are considered to be an important characteristic of human cancer. The progress of valuable and successful cancer therapies depends mainly on the recognition of physiologic targets that are primarily involved in the regulatory mechanism of cell cycle progression [1–3]. The members of serine/threonine kinases, such as cyclic-dependent kinase, polo-like kinases, aurora kinases, and NIMA-related kinase (Nek) are the well-studied families that coordinate the mitosis sequence [4]. Many studies are generally focused on the development of inhibitors for these mitotic kinases and efforts have been put up to utilize cell cycle targets for generation of new anticancer drugs [5, 6].

In recent years, the members of NIMA-related kinases (Nek) family have contributed to various aspects of mitotic progression and cytokinesis [7]. In mammals, about eleven types of NIMA-related kinases are recorded and designated as Nek1 to Nek11, which all structurally share a conserved N-terminal catalytic domain, pursued by a nonconserved C-terminal regulatory domain. However, Nek6 and Nek7 are smaller molecules and consist only of a catalytic domain with a moderately short N-terminal expansion. The functions of Nek6 and Nek7 are concerned with the control of mitotic spindle formation, mutually with the upstream Nek9 in a mitotic kinase cascade [8]. Even though Neks exhibita major role in cell cycle related functions, their mechanism of regulation remains unclear [9]. The tumorigenesis role of Nek6 is well established through several lines of evidence. The increased levels of Nek6 protein expression and kinase activity have recently been accounted in a diverse range of malignant cancers like breast, colon, lung, and gastric cancers [10]. Furthermore, it has been shown that the over expression of Nek6 can endorse cell alteration while suppression of Nek6 resulted in inhibition of anchorage-independent growth and stirred apoptosis in most cancer cells. A recent report suggests that during premature senescence, Nek6 expression levels are diminished and the ectopic execution of Nek6 prevents p53-induced premature senescence of human cancer cells [11]. Nek6 is regarded as a direct target of the DNA damage checkpoint and the inhibition of Nek6 function is necessary for G_2_/M arrest in the lead of DNA damage [12]. Hence, Nek6 has emerged as a therapeutic target for drug development towards cancer [5].

In this context, we elucidate the structural information of Nek6, which may be a new drug target for developing inhibitors against cancers by means of a homology modeling approach pursued by a molecular dynamic simulation in order to explore the stability of the protein. However, to date, the structural or drug targeting information against human Nek6 is unavailable. In addition, we calculated the binding site of protein to identify drug-like molecules that acquire enhanced binding energies and pharmacokinetic properties for this Nek6 through high throughput virtual screening. Therefore, the drug-like molecules through screening procedure may act as novel leads for Nek6 inhibitors designing.

## 2. Materials and Methods

### 2.1. Homology Modeling of Human Nek6 Protein

Homology modeling is an efficient method for 3D structure prediction and quick experimental design for docking studies. The crystal structure of Nek6 protein is currently unavailable in the Protein Data Bank (PDB). Hence, homology modeling studies have been conducted based on high-resolution crystal structures of homologous proteins. The Nek6 protein sequence was retrieved from Uniprot (Accession no. Q9HC98) and it contains 313 amino acid residues. A sequence similarity search for the protein against other sequences with available structural information was performed using the NCBI BLAST. Crystal structure of APO human Nek7 (PDB ID: 2WQM with 2.10 Å resolution) was selected as template, having 82% sequence identity with target. The homology molecular modeling and model validation were performed in a similar way to that described in Meirelles et al., 2011 [4]. The Nek6 protein was modeled by using SWISS-MODEL [13], MODELLER 9v8 [14], and Prime [15] and the modeled protein was further validated.

### 2.2. Structural Validation

The constructed Nek6 structure was validated by the inspection of phi/psi distributions of Ramachandran plot obtained through PROCHECK analysis [16]. The quality of Nek6 structure was further analyzed by ERRAT program [17]. The significance of consistency between template and modeled Nek6 was evaluated using ProSA server [18]. In addition, the root mean square deviation (RMSD) was analyzed by 3D-ss server [19] on superimposition of template (2WQM) with predicted structure of Nek6 to check the reliability of the model.

### 2.3. Molecular Dynamic Simulation

The molecular dynamics (MD) simulation of modeled protein was performed using the program Desmond v 3.0 as implemented in Schrödinger package [20]. Initial minimization of model was done to discard high energy intramolecular interaction by using OPLS (2005) force field [21], and SPC (single point charge) system was used to mimic the physiological behavior of the molecules. The simulation was carried out with the default protocol provided in Desmond, which consists of a series of restrained minimizations and molecular dynamics simulations that are designed to slowly relax the system without deviating considerably from the initial protein coordinates. The SPC water molecules were added, the boundary condition was set as orthorhombic, and the system was neutralized by adding Cl^−^ counter ions to balance the net charge of the system. Prior to the MD simulations, 2,000 steps of minimization with the steepest descent method were used to remove high-energy interatomic contacts. Finally, molecular dynamic simulation was carried out at 300 k for 3 ns of simulation. The RMSD and energy value were calculated to study the structural alterations and dynamic deeds of the protein.

### 2.4. Active Site Prediction

The active sites (binding pockets) and functional residues of Nek6 were identified and characterized by Site-Map module from Schrodinger package [22]. Site-Map calculation begins with an initial search step that identifies or characterizes, through the use of grid points, one or more regions on the protein surface that may be suitable for binding ligands to the receptor. Contour maps were then generated, produced hydrophobic, hydrophilic maps hydrogen binding possibilities [23] which may guide the protein-ligand docking analysis.

### 2.5. Virtual Screening Approach

The virtual screening workflow (Maestro, Schrödinger, 2009) [24] was used to identify the potential small molecule inhibitors against putative active site of Nek6 protein. A total of 2, 84, 206 small molecules obtained from the Binding database (http://www.bindingdb.org), TOS Lab Collection (http://www.toslab.com/), and MayBridge databases (http://www.MayBridge.com/) were carried out through high throughput virtual screening (HTVS). The protocol constraints such as Run QikProp, Lipinski filter, and Reactive filter were set to filter ligands with suitable pharmacological property and no reactive functional group [23]. Glide HTVS, Standard Precision, and Extra Precision parameters were checked and set to save 20% of the good scoring ligands. Finally, top most 10 lead compounds were screened based on Glide score, Glide energy, and hydrogen bond interactions and best hit compounds were selected for IFD analysis.

### 2.6. Induced Fit Docking Studies

A mixed molecular docking/dynamics protocol, called induced-fit docking (IFD) was performed by the following steps [25]. (i) The ligand was docked into a rigid receptor model with scaled down vdw radii (Glide SP mode). A vdw scaling of 0.5 was used for nonpolar atoms of both Nek6 and lead compounds from HTVS. (ii) The prime induced fit with refined residues within 3.4 Å of ligand poses was carried out. (iii) The Glide redocking analysis was performed. Ten initial ligand poses of Nek6 structure were selected and refined, and induced-fit protein-substrate complexes were generated by Prime. The side chain and backbone structures of amino acid residue within 5.0 Å of Nek6 were refined and they were ranked by Prime energy. The protein-substrate interaction energy and the total energy were calculated by OPLS-2005 force field with implicit solvation model for ranking the IFD poses [26].

## 3. Results and Discussion

### 3.1. Homology Modeling of Nek6 Protein and Its Validation

**Figure 1:**
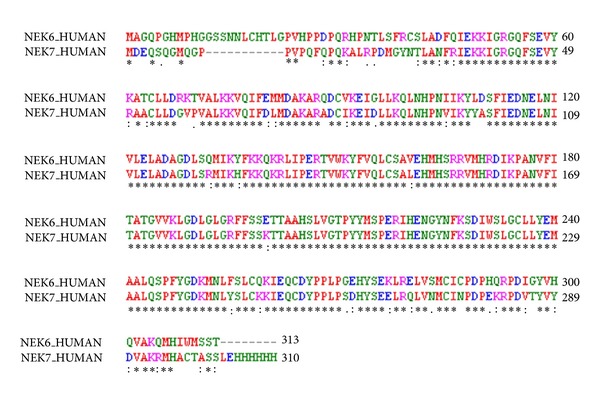
Sequence alignment of Nek6 (target) with Human APO Nek7 protein (template). Asterisks indicate identical amino acids, dots indicate similar amino acids.

**Figure 2:**
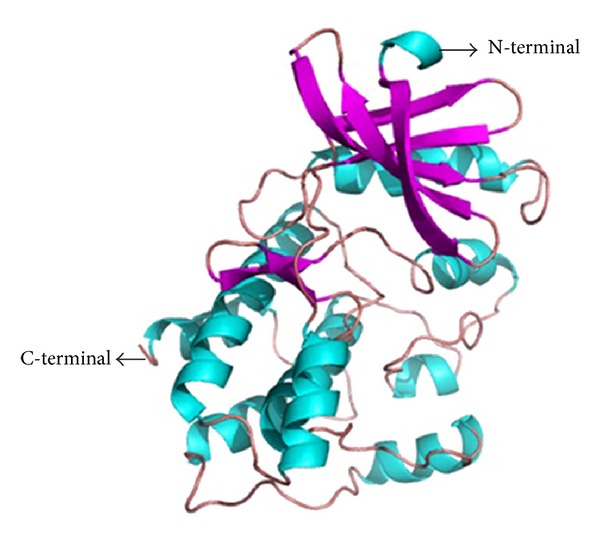
Three-dimensional (3D) structure of Nek6 based on known template protein structure. The structure is shown in secondary structure mode using Pymol.

Homology modeling is usually the method of choice when a clear relationship of homology between the sequence of target protein and at least one known structure is found. This approach would give reasonable results based on the assumption that the tertiary structures of two proteins will be similar if their sequences are related [27]. The absence of three-dimensional structure of Nek6 motivated us to construct this model. Accordingly, the crystal structure of Human APO Nek7 is an appreciated template for modeling the 3D structure of Nek6. The sequence alignment of Nek6 with human APO Nek7 portrays that both template and target share significant similarity with each other (~86% identical), within their catalytic domains (Figure 1). Several homology modeling tools were used to model the protein, and comparison of the results indicated that the model generated by SWISSMODEL is more acceptable than those generated by the other programs (more amino acids in the most favourable regions and less in the disallowed regions). The structure prediction results were very much similar to those described by Meirelles et al., 2011 [4]. The primary 3D model of Nek6 resulted from the structural modeling is shown in Figure 2.

**Figure 3:**
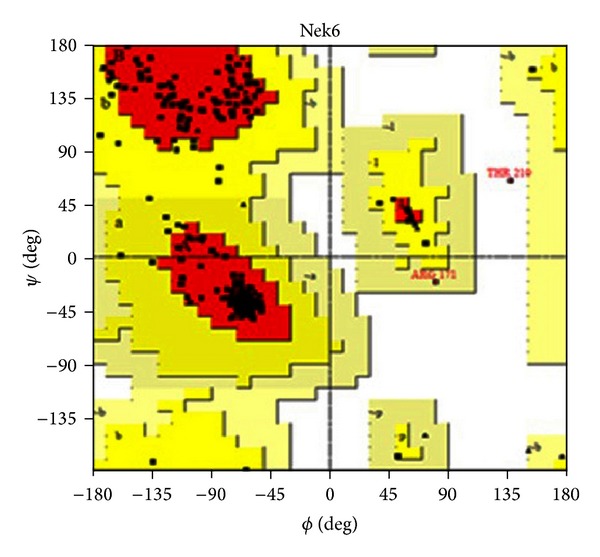
The stereochemical spatial arrangement of amino acid residues in the modelled 3D structure of Nek6 in the favoured region of the Ramachandran plot.

**Figure 4:**
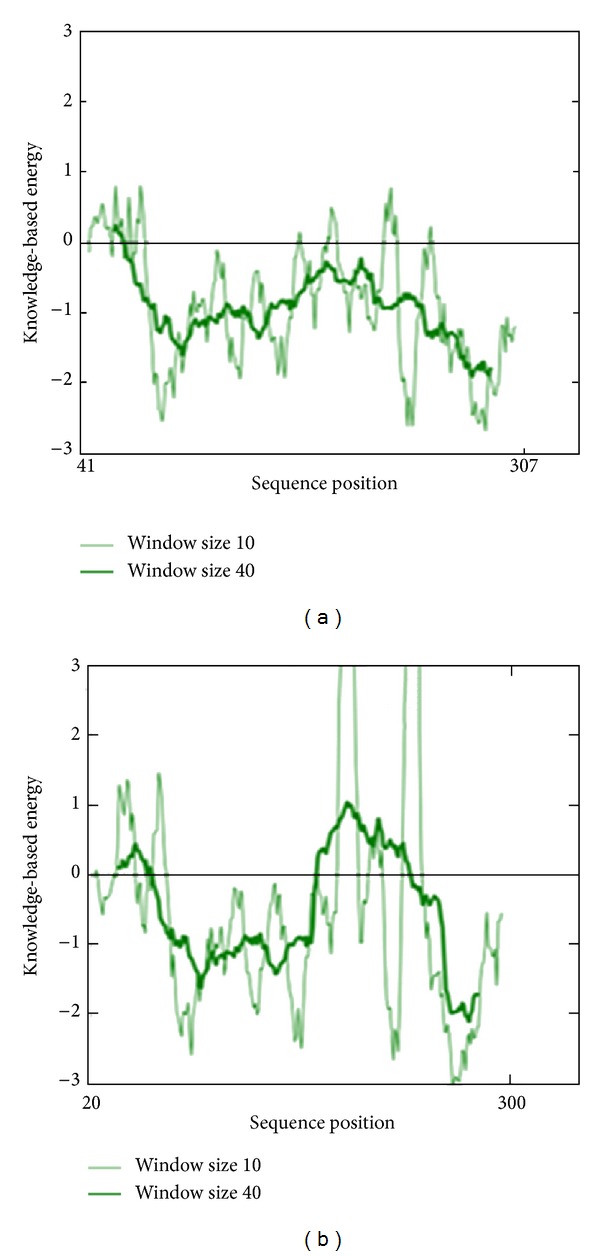
Prosa energy profiles calculated for Nek6 model (a) and the Nek7 crystal structure (b).

**Figure 5:**
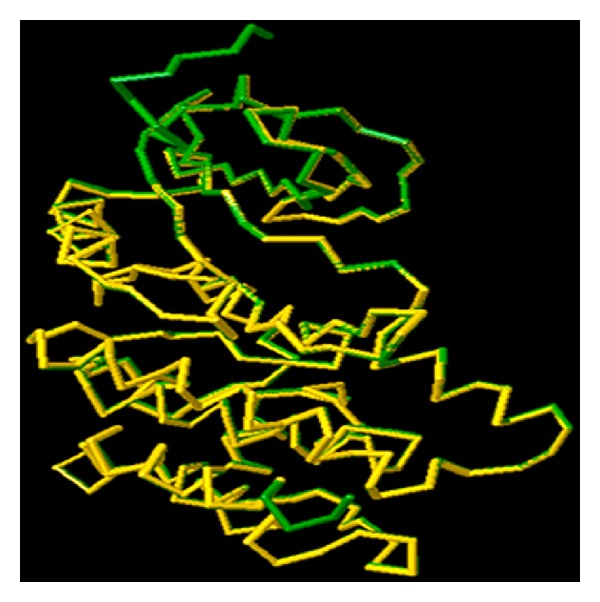
Superimposition of modeled Nek6 (target) on Nek7 (template) using the 3d-SS tool. In this wireframe diagram, yellow represents the target and green represents the template.

The backbone conformation of the refined model was validated using Ramachandran plot obtained through PROCHECK. The distribution of the phi and psi angles for the amino acid residues was represented by Ramachandran plot. The generated model was found to be highly plausible; hence, 0.4% (only one residue) of residues alone was found to span the disallowed region of Ramachandran plot (Figure 3). The Ramachandran plot residues of Nek6 and 2WQM template (Nek7) were compared for validation (Table 1). The measurement of the structural error at each amino acid residue in the 3D structural model was given by the ERRAT plot. The overall quality factor of the computed model was 89.57% (see the Supplementary Material available online at http://dx.doi.org/10.1155/2014/967873, Figure S1). Evaluation of Nek6 (target) 3D model and Nek7 (template) with ProSA server revealed a compatible *Z* score value of −7.59 and −5.99, respectively. This shows that residue energies together with pair energy, combined energy, and surface energy were all negative and had comparable surface energy affinity with template. The *Z* score of modeled Nek6 and human APO Nek7 protein is illustrated in Figure 4. The RMSD was calculated between the target and template structure and it was found to be 0.782 Å (Figure 5). The low overall RMSD reflects high structural conservation making it a good system for homology modeling [28]. Hence, the predicted model which proved to be well ratified in terms of geometry and energy contours proposed that the model is reasonable and reliable for further molecular docking studies [29, 30].

### 3.2. Molecular Dynamic Study

**Figure 6:**
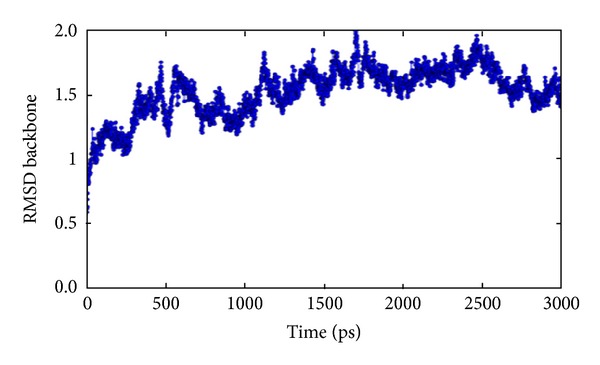
Calculated root mean square deviation (RMSD) graph of backbone atoms of Nek6. *X*-axis: Time (ps), *Y*-axis: RMSD.

To gain insight into the stability and dynamic properties of the homolog structures, explicit solvent MD simulation is performed [31]. The verified and restrained Nek6 model was used for molecular dynamic studies. The hydrogen atoms were added, equivalent to the experimental pH condition in order to mimic the protein structure. Furthermore, to relax the close contact conformation, the added hydrogen atoms were energy minimized. The salt concentration of 0.15 M was prepared with the help of Na^+^ and Cl^−^ ions. The simulation was carried out for 3 ns at 300 K temperature in MTK_NPT ensemble class. The overall structural fluctuation was evaluated by analyzing the RMSDs of backbone atom versus simulation time. The potential energy, temperature, pressure, and volume were evaluated during MD simulation (Supplementary Figures S2(a)–S2(d)). The protein structure reached equilibrium after 500 ps and maintained equilibrium below 2.0 Å up to 3000 ps, indicating that the system evolved into stable states and was reasonably converged. In general, the trajectories produced a stable protein with an average RMSD value of about 2.0 Å (Figure 6).

### 3.3. Active Site Analysis

The best active site (binding pocket/site) was preferred based on the site score and hydrophobic/hydrophilic areas, which holds better binding cavity. The binding site residues of modeled Nek6 predicted by Sitemap yields 16 amino acid residues in the binding pocket, namely, Ile51, Arg53, Gln55, Lys74, Glu123, Ala125, Asp129, Ser131, Lys135, Lys174, Asn177, Asp190, Gly192, Thr210, Tyr212, and Ser245 (Supplementary Figure S3). These binding site residues may be involved in the binding of substrate and small molecule inhibitors. Thus, all these residues were confirmed as Nek6 active site residues and picked to generate grid in the centroid of these residues for virtual screening approach [28]. The C-terminal kinase domain of Nek6 protein consists of two important key signatures which includes ATP-binding and Ser/Thr kinases active-site regions, more or less similar to Nek7 protein. The ATP-binding pocket of Nek6 consists of 23 amino acid residues that fall between Ile51 and Lys74 [4], and most inhibitors of kinases were developed against ATP binding site [32]. Hence, in this study, the predicted binding site residues of Nek6 also include certain residues that belong to ATP-binding pocket.

### 3.4. Virtual Screening and IFD Studies

One of the most widely used methods for virtual high throughput screening is docking of small molecules into active site of protein target and subsequent scoring. The Binding database was screened against the putative active site of Nek6 protein using virtual screening work flow (Maestro, Schrödinger, 2009). The ligands were prepared at pH 7.0 ± 2.0 using Epik state and the large penalties of high energy ionization or tautomer states were removed. The protein was kept as scaling van der Waals radius by 1.0 Å and partial atomic charge that is less than 0.25 Å at default constraint parameters. The Glide HTVS was performed with flexible docking algorithm using selected constraints for each grid in OPLS 2005 force field [26]. Using Glide HTVS, about 8,750 compounds were screened from Binding, TOS Lab, and MayBridge database based on their pharmacokinetic properties and ability to bind into the active site region of Nek6 protein. Further, screening of these compounds in Glide XP mode results in 35 compounds which have higher binding affinity towards Nek6. Based on Glide XP results, ten top most compounds (all resultant from Binding database) were identified against Nek6 protein (Table 2).

**Figure 7:**
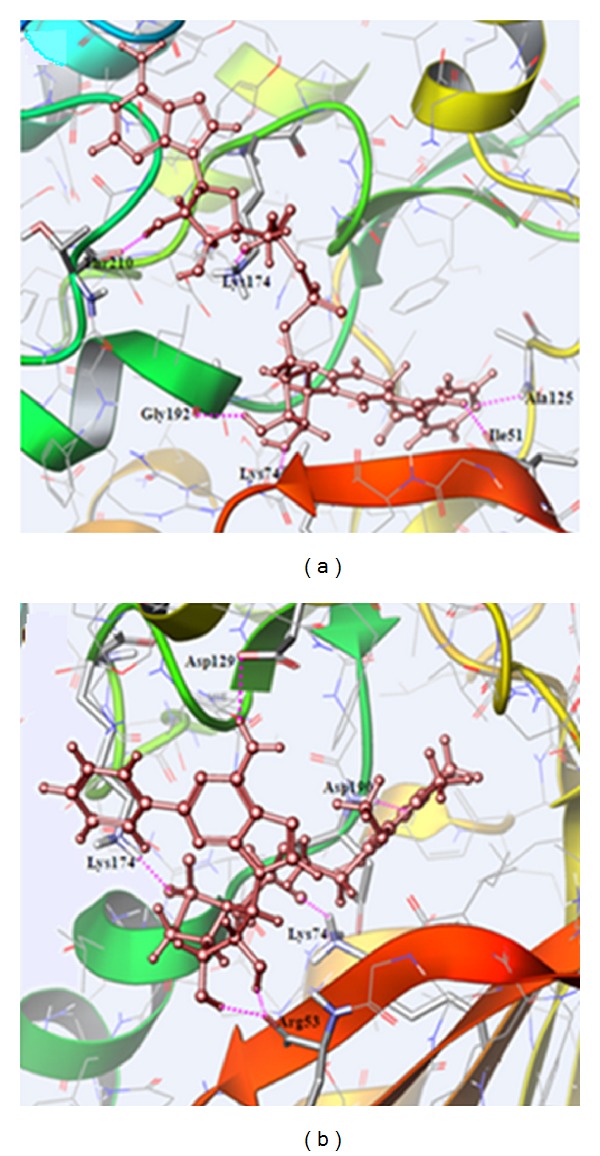
The binding mode of compounds (a) 15666 and (b) 18602 with Nek6 obtained from virtual screening approach. Hydrogen bonds are shown in dotted pink lines.

**Figure 8:**
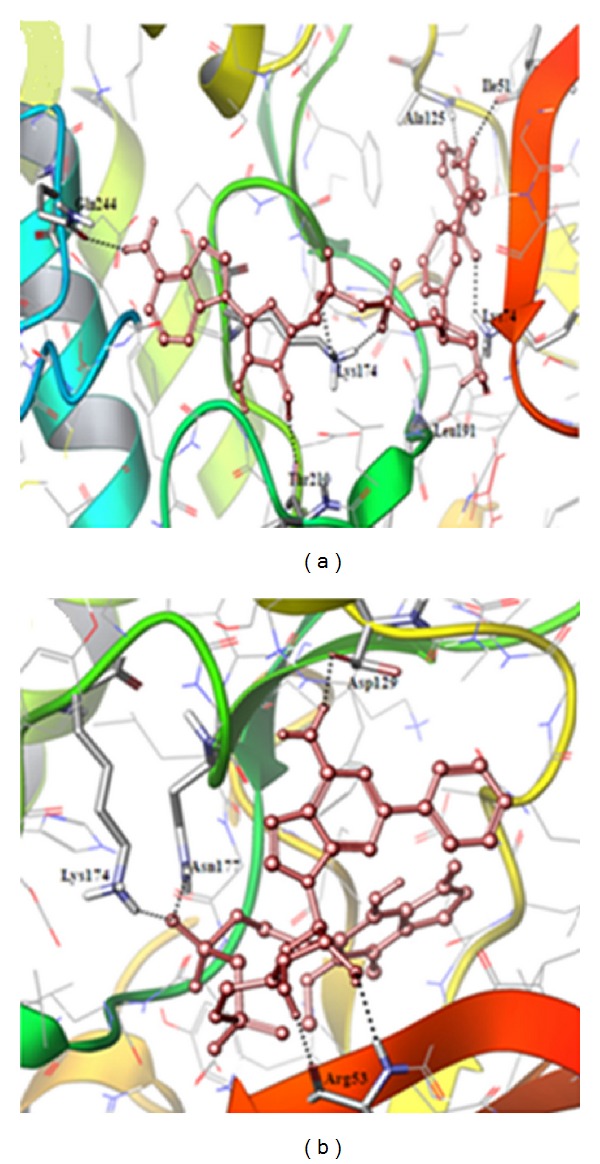
The binding mode of compounds (a) 15666 and (b) 18602 with Nek6 obtained from IFD approach. Hydrogen bonds are shown in dotted black lines.

Among the ten compounds, the compounds ID 15666 and 18602 have higher binding affinity with high Glide score of −12.024, −11.095 and favorable Glide energy of −69.773, −70.845 kcal/mol, respectively. The compounds id 15666 and 18602 were generally known as INH-NAD Adduct and Mycophenolic Adenine Dinucleotide (MAD) Analogue, 37. The docked protein-ligand complexes with hydrogen bond interactions are shown in Figure 7 and they possess multiple hydrogen bond interactions with the active site residues. Mostly, the lead compounds exhibit their interactions within the ATP-binding pocket through Gln55, Arg53, Ile51, and Lys74 residues. In search of biological active data of the top two lead compounds (id 15666 and 18602), it was noted that they do not exhibit any activity towards other kinases. But, the compounds were proved to demonstrate inhibitory activity towards enoyl-ACP reductase of *Mycobacterium tuberculosis* (id 15666) [33] and inosine monophosphate dehydrogenases, a possible target for cancer chemotherapy (id 18602) [34]. Subsequently, the top 10 compounds selected from Glide XP docking studies were analyzed by a mixed molecular docking/dynamic approach (IFD) [25]. The results originated through IFD are exemplified in Table 3 and in most cases, the glide scores were very close to Glide XP. The binding mode of the compounds (id 15666 and 18602) with Nek6 through IFD approach is illustrated in Figure 8.

**Figure 9:**
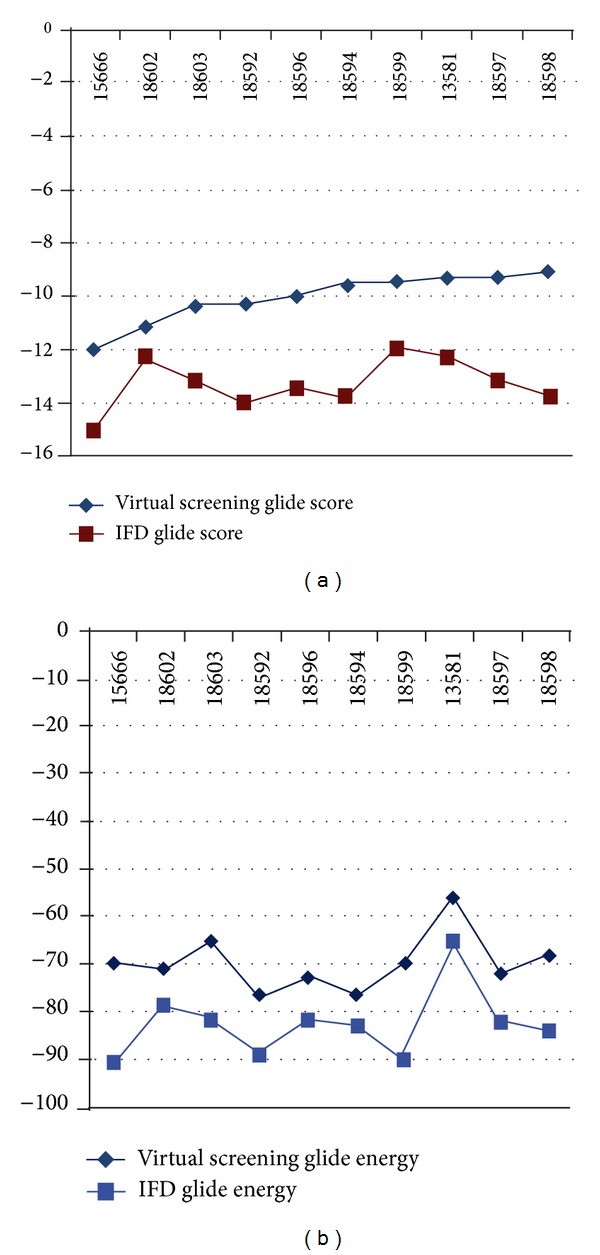
The graphical comparison of Glide score (a) and Glide energy (b) of ten lead compounds obtained through virtual screening and IFD approach.

The comparison of Glide XP results obtained through virtual screening and IFD for the compound (id 15666) clearly shows that they possess less glide score of −12.024 when compared to IFD glide score (−14.934). Similarly, the compound (id 18602) also exhibits low Glide XP score (−11.095) when compared to IFD (−13.395) score, respectively. The glide energy also varies from −69.773 kcal/mol to −90.700 kcal/mol for both compounds. The scores and energies obtained from both studies for all ten compounds are shown in Figure 9. The comparison of results obtained through virtual screening and IFD sheds a new light that all compounds interact in a more or less similar binding pattern with Nek6 protein. In addition, we observed that the amino acid Lys174 plays a major role in receptor-ligand interaction of all ten compounds during both of the docking procedures. Hence, we hypothesize that the compounds (id 15666 and 18602) can be tested for their biological activity through *in vivo*/*in vitro* studies for the development of drug candidate against Nek6 protein.

## 4. Conclusion

In this study, we have developed a homology model of Nek6 based on known crystal structure of Human APO Nek7 protein, as expected from its sequence similarity. After the energy minimization, molecular dynamics simulation was carried out for the modeled structure which results in the most reliable model and this stable structure is further used for receptor ligand interaction analysis. Using Binding database, the high throughput virtual screening was performed which resulted in 10 hit compounds against Nek6 protein. The Glide XP and IFD docking simulations reveal that each compound was bound to the similar region on the active site of Nek6 protein. Hence, we reported that the compounds 15666 and 18602 were good inhibitors against Nek6 protein through its Glide score and Glide energy and will be helpful for the development of drug candidate against Nek6 protein.

## Supplementary Material

Supplementary Figure S1: Errat value of Nek6 model built using NCBI Structural Analysis and Verification Server
Supplementary Figure S2: ^a^Potential energy (kcal/mol) during 3000 ps of molecular simulation. ^b^ The pressure (bar) during 3000 ps of molecular simulation, ^c^ temperature (K) during 3000 ps of molecular simulation, and ^d^ the volume (A) during 3000 ps of molecular simulation.
Supplementary Figure S3: Active site (pink color) of the Nek6 protein

## Figures and Tables

**Table 1:** Percentage residues of Nek6 and Nek7 proteins predicted by Ramachandran plot statistics.

Statistics	Percentage of residues in Nek6 (target)	Percentage of residues in Nek7 (template)
Residues in most favored region	87.4	90.8
Residues in additionally allowed region	11.7	8.8
Resides in generously allowed region	0.4	0.4
Residues in disallowed region	0.4	0.0

**Table 2:** Extra Precision (XP) results for the ten lead compounds obtained through virtual screening approach.

S. no.	Lead molecules^a^	Glide score	Glide energy (kcal/mol)	H-bond interactions	Interacting amino acids
1	15666	−12.024	−69.773	6	Ile51, Lys74, Ala125, Lys174, Gly192, Thr250
2	18602	−11.095	−70.845	6	Arg53, Arg53, Lys74, Asp129, Lys174, Asp190
3	18603	−10.357	−65.248	6	Gln55, Lys74, Lys174, Asn177, Asp190, Asp190
4	18592	−10.189	−76.731	5	Arg53, Lys174, Lys174, Tyr212, Ser245
5	18596	−10.006	−72.922	8	Ile51, Ile51, Lys74, Glu123, Ala125, Ser131, Lys135, Lys174
6	18594	−9.560	−76.319	6	Ile51, Lys74, Glu123, Alu125, Lys174, Asn177
7	18599	−9.477	−69.921	7	Arg53, Arg53, Lys74, Ala125, Asp129, Lys175, Asn177
8	13581	−9.333	−56.474	4	Lys74, Lys135, Lys174, Asp190
9	18597	−9.253	−72.195	7	Ile51, Arg53, Lys74, Glu123, Ala125, Lys174, Asn177
10	18598	−9.040	−68.177	4	Lys74, Ala125, Lys174, Asp190

^a^Ligand IDs are from the Binding database.

**Table 3:** Summary of IFD results for ten best lead molecules.

S. no.	Compound ID	Glide score	Glide energy (kcal/mol)	IFD score	Interaction amino acids
1	15666	−14.934	−90.700	−609.466	Ile51, Ala125, Lys174, Leu191, Lys74, Thr210, Gln244
2	18602	−12.262	−78.495	−601.331	Arg53, Lys174, Asn177, Asp129
3	18603	−13.177	−81.516	−603.036	Asp129, Arg53, Lys174, Leu193, Lys74, Ala125
4	18592	−14.058	−89.051	−605.962	Gln55, Arg53, Ile51, Lys74, Ala176, Asn177, Ala125
5	18596	−13.395	−81.759	−606.784	Pro211, Lys174, Gly192, Ser57
6	18594	−13.828	−83.107	−606.325	Ala176, Lys174, Ala125, Arg53, Lys74
7	18599	−11.869	−90.037	−605.996	Thr210, Lys174, Asn177, Lys74, Arg53, Ile51, Glu123
8	13581	−12.211	−65.306	−604.068	Asp126, Ala127, Asp129, Asp190
9	18597	−13.041	−82.403	−605.047	Tyr212, Thr210, Lys174, Lys74, Asp190, Glu123
10	18598	−13.709	−84.140	−606.432	Lys174, Leu193, Arg53, Ile51, Ala125
