# Supplementary material for: Discovery of Novel Inhibitors for Nek6 Protein through Homology Model Assisted Structure Based Virtual Screening and Molecular Docking Approaches
Source: ScientificWorldJournal. 2014 Jan 22;2014:967873. doi: 10.1155/2014/967873 (PMC3920677; doi:10.1155/2014/967873)
Supplement: Supplementary file 1 — Supplementary Figure S1: Errat value of Nek6 model built using NCBI Structural Analysis and Verification Server Supplementary Figure S2: aPotential energy (kcal/mol) during 3000 ps of molecular simulation. b The pressure (bar) during 3000 ps of molecular simulation, c temperature (K) during 3000 ps of molecular simulation, and d the volume (A) during 3000 ps of molecular simulation. Supplementary Figure S3: Active site (pink color) of the Nek6 protein [file 967873.f1.doc]

**Supplementary Figure S1:** Errat value of Nek6 model built using NCBI Structural Analysis and Verification Server


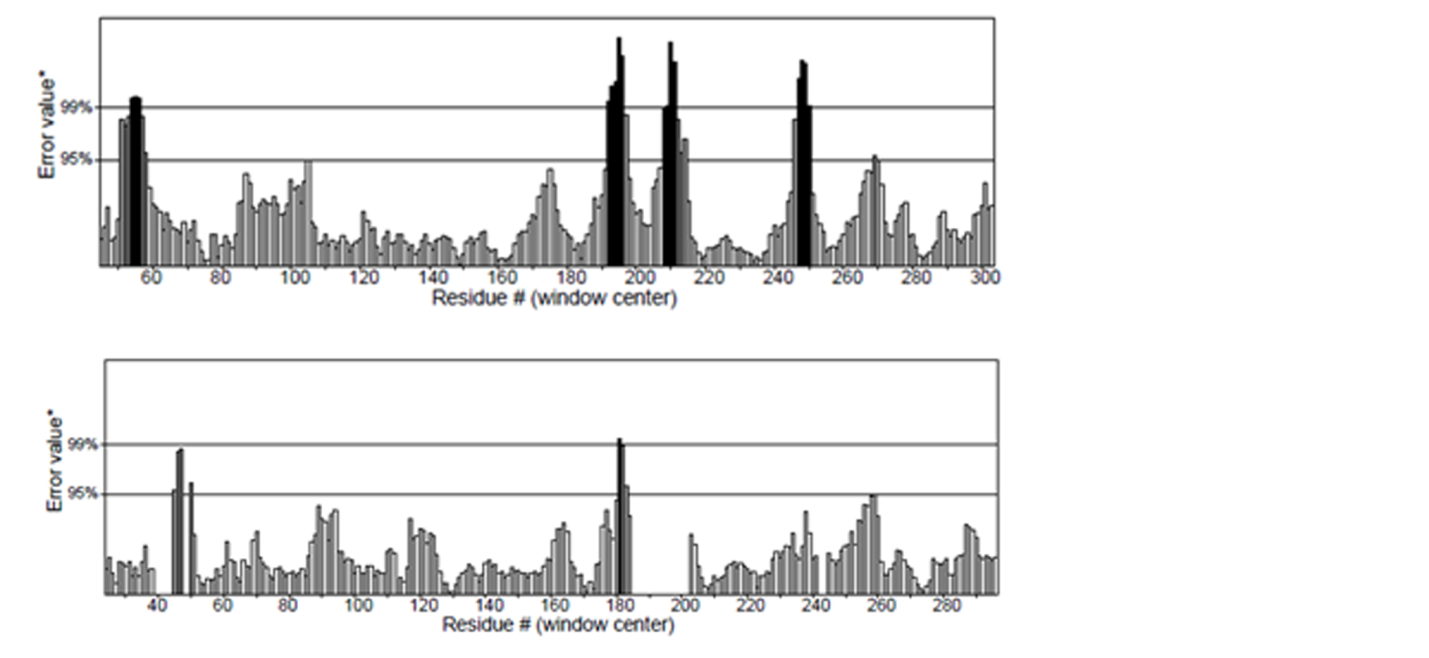


**Residue # (Window Center) Glide Score**

60 80 100 120 140 160 180 200 220 240 260 280 300

**Error Value**

99%

95%

**Supplementary Figure S2:**  ^a^ Potential energy (kcal/mol) during 3000 ps of molecular simulation. ^b^ The pressure (bar) during 3000 ps of molecular simulation, ^c^ temperature (K) during 3000 ps of molecular simulation, and ^d^ the volume (A) during 3000 ps of molecular simulation.


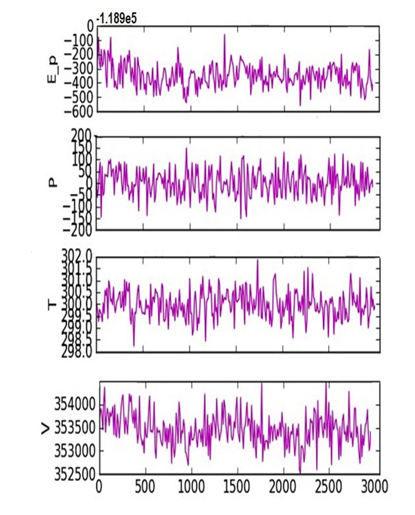


**-1.189e5**

**E_p**

0

-100

-200

-300

-400

-500

-600

**a)**


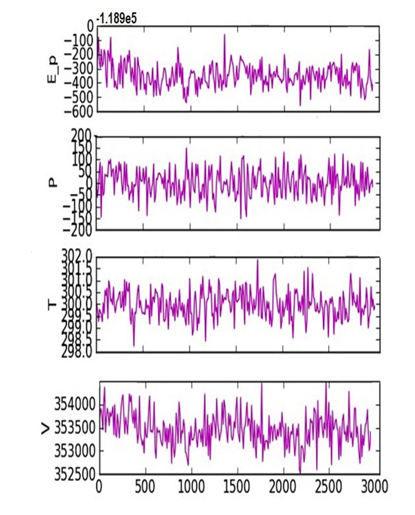


**P**

200

150

100

50

0

-50

-100

-150

-200

**b)**


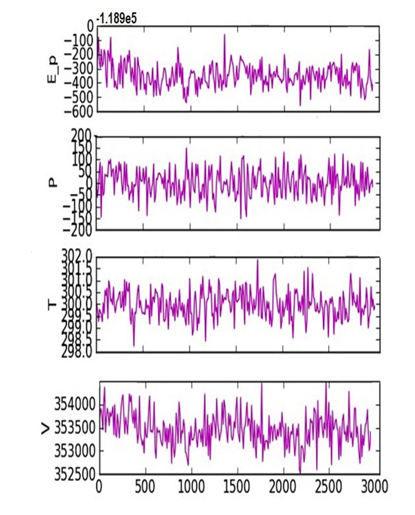


**T**

302.0

301.5

301.0

300.5

300.0

299.5

299.0 298.5

298.0

**c)**


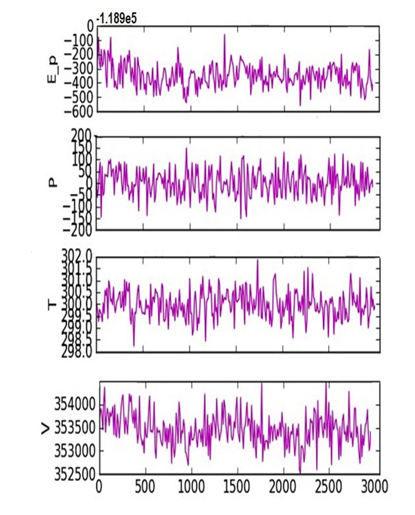


0 500 1000 1500 2000 2500 3000

**V**

354000

353500

353000

352500

**d)**

**Supplementary Figure S3:** Active site (pink color) of the Nek6 protein


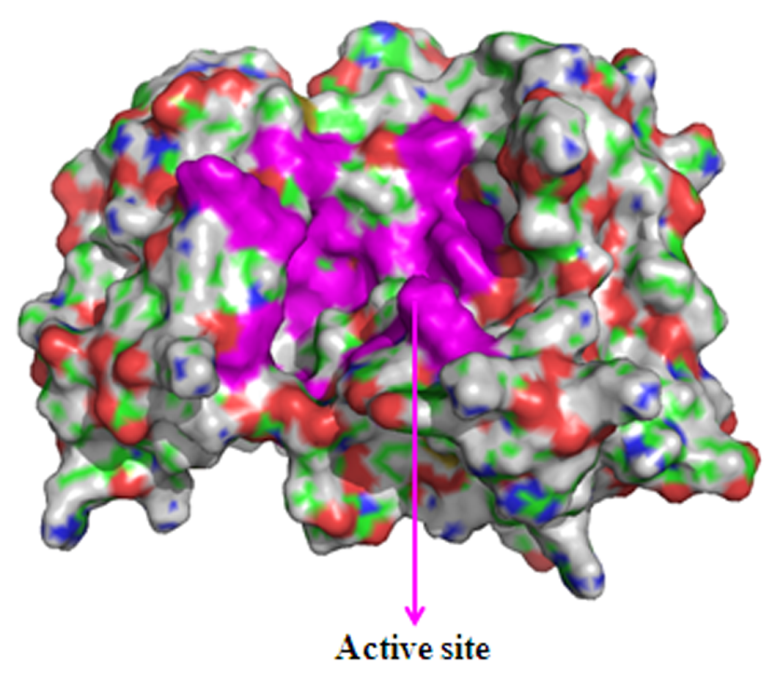


**Active Site**
